# IgG4-Related Diseases–Continues To Be a Cancer Mimicker

**DOI:** 10.7759/cureus.6610

**Published:** 2020-01-09

**Authors:** Soujanya Sodavarapu, Gurinder S Ghotra, Nashwan Obad, Megha Goyal, Amandeep S Gill

**Affiliations:** 1 Internal Medicine, San Joaquin General Hospital, French Camp, USA; 2 Internal Medicine, San Jaoquin General Hospital, French Camp, USA; 3 Hematology-Oncology, San Joaquin General Hospital, French Camp, USA

**Keywords:** pancreatic mass, igg4-related disease

## Abstract

Immunoglobulin G4-related disease (IgG4-RD) is a chronic multisystem immune-mediated disease. Histologically, it presents as infiltration with IgG4-secreting plasma cells affecting many organs such as the pancreas, lacrimal glands, salivary glands, kidneys, and arteries, resulting in chronic fibrosis and scarring within the tissue which over time forms into a mass. It is a slow-growing process that usually remains indolent until it causes a mass effect or tissue infiltration.

Our patient was found to have a pancreatic mass on imaging concerning pancreatic neoplastic lesions. He subsequently underwent biopsy for histology/pathology and IgG4 levels, which led to our diagnosis of IgG4-RD. Imaging, blood tests, and, most importantly, histopathological features of the involved organ are important in determining the diagnosis. This is important as a treatment plan will be based on the given diagnosis, and patients with IgG4-RD respond very well to systemic steroid therapy.

## Introduction

Immunoglobulin G4-related disease (IgG4-RD) is a multisystem chronic mass-forming inflammatory condition characterized by tissue infiltration with lymphocytes and IgG4-secreting plasma cells, storiform fibrosis, and obliterative phlebitis histologically [1]. Diagnosis of IgG4-RD can be challenging as it leads to the formation of a “mass” within organs often misdiagnosed as malignancies. It can involve one or multiple organs, with the most commonly affected organs being the pancreas, lacrimal/salivary glands, and the kidneys. It is an indolent condition, often incidentally diagnosed on imaging studies, but at times it can cause significant symptoms mostly from obstruction or compression of nearby structures due to the mass effect. It is crucial to keep IgG4-RD in mind when dealing with “mass-like” etiology [1].

## Case presentation

A 60-year-old male with a history of cryptogenic liver cirrhosis and newly diagnosed diabetes presented with abdominal discomfort and unintentional weight loss for a few months.

The patient’s physical examination showed mild diffuse abdominal pain, otherwise unremarkable. His labs showed a slight elevation in alkaline phosphatase, mild elevation in globulin level at 4.3 g/dL, normal C-reactive protein, and mild elevation in erythrocyte sedimentation rate of 28 mm/hr. Alpha-fetoprotein, carcinoembryonic antigen, and CA 19-9 levels were normal. PPD and QuantiFERON gold were negative; antinuclear antibody, anti-smooth muscle antibody, and antimitochondrial antibody levels were also normal; hepatitis panel was negative. He underwent further imaging, including computed tomography (CT) with contrast scan of the abdomen, which showed the following: a poorly defined 4.5 cm mass in the pancreatic body encasing the splenic artery, indicative of pancreatic adenocarcinoma; mild intrahepatic bile duct dilatation, primarily on the left liver lobe, with circumferentially thickened gallbladder wall; a 2.6 cm mass at the deep margin of the umbilicus possible rectal cyst vs. metastasis; or postsurgical changes. It showed soft tissue thickening anterior to the distal abdominal aorta and encasing of inferior mesenteric artery suspicious for possible metastatic disease or chronic post-inflammatory changes. The patient was furthered referred to oncology for evaluation.

**Figure 1:**
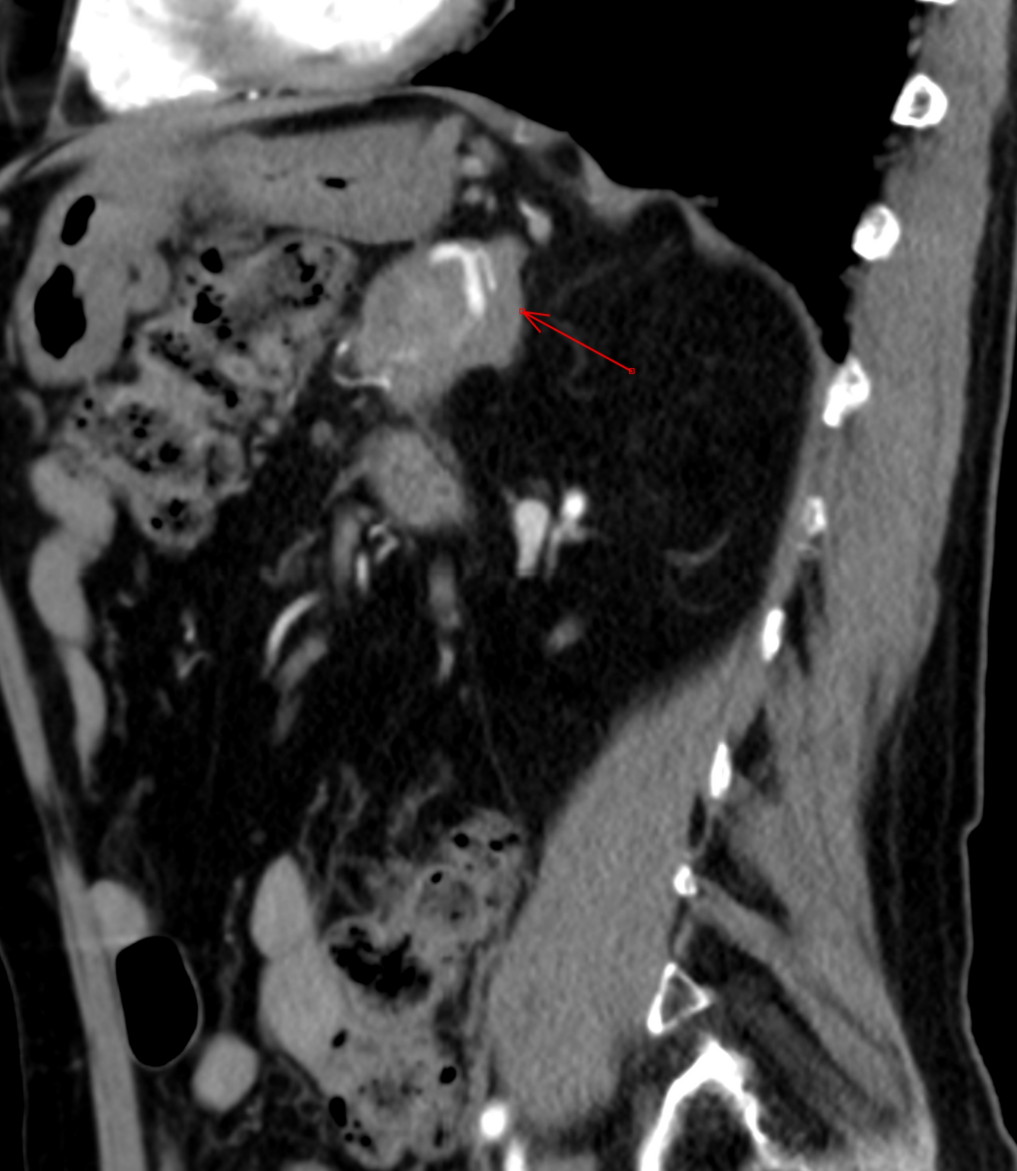
Computer tomography with contrast of the abdomen and pelvis showing pancreatic mass encasing splenic artery

**Figure 2:**
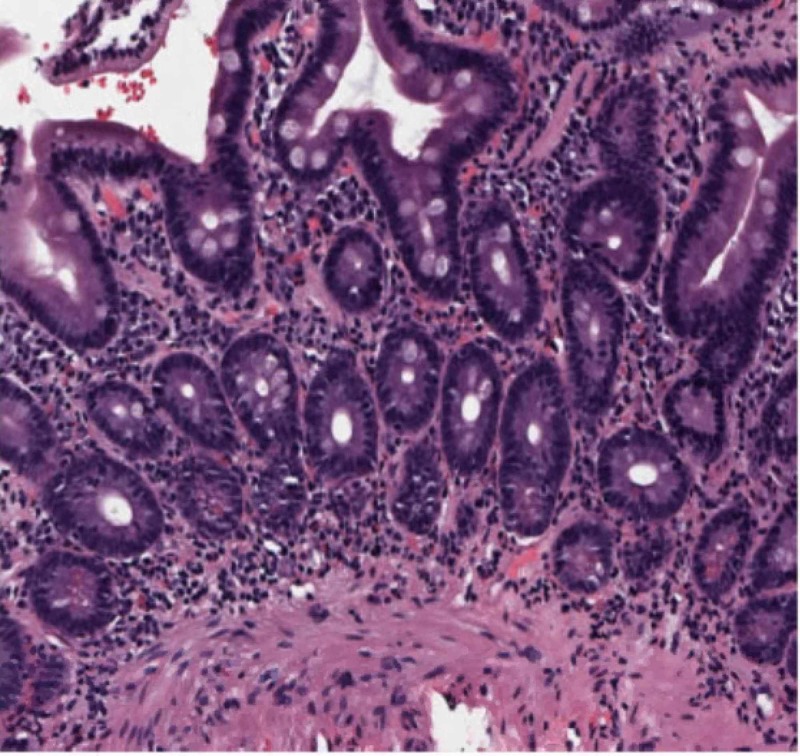
Hematoxylin and eosin stain of duodenal biopsy showing plasma cells

**Figure 3:**
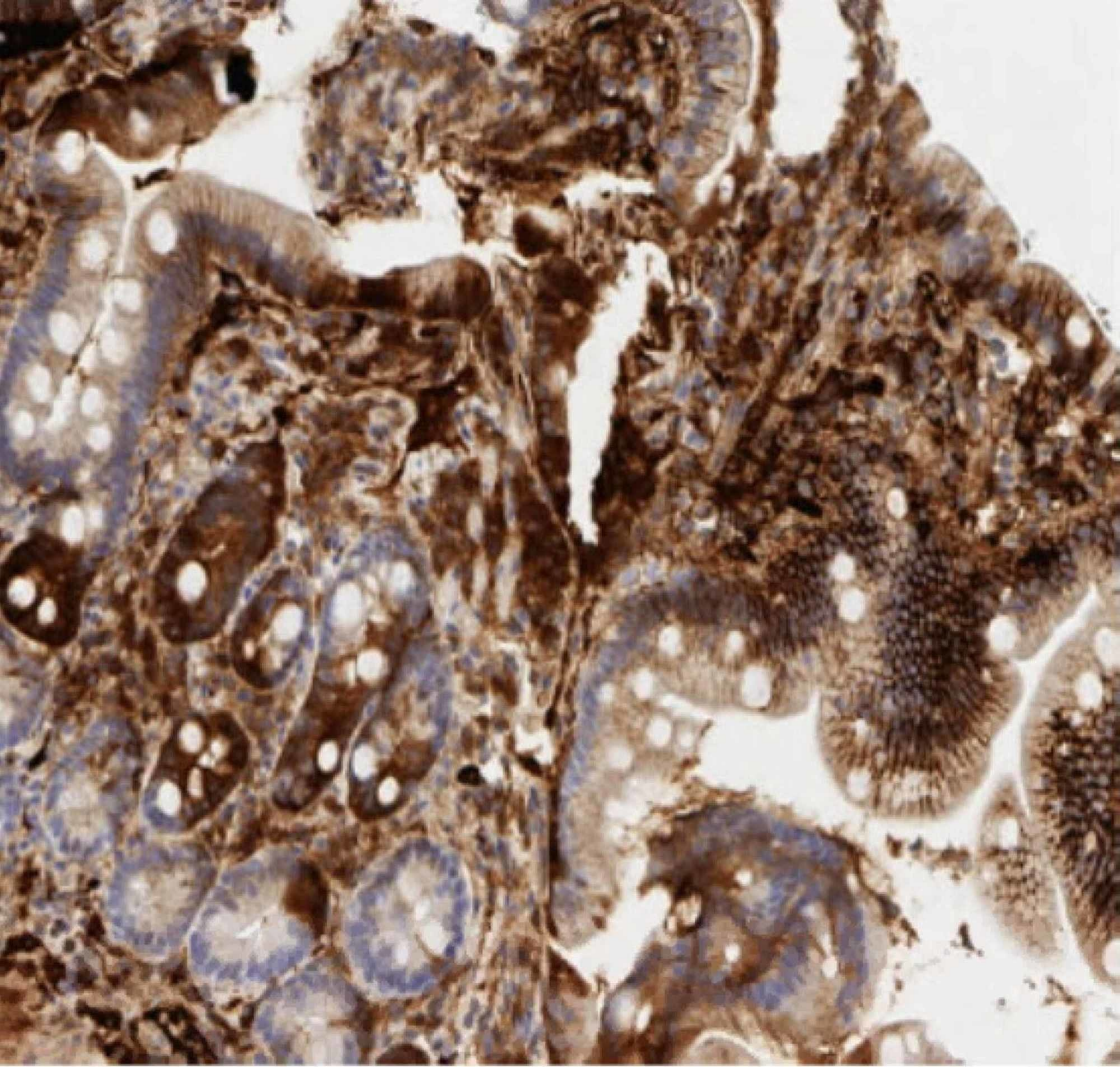
IgG4 stain positive on the duodenal biopsy

Our patient then underwent esophagogastroduodenoscopy, which showed two columns of trace distal esophageal varices with no stigmata for bleeding; no bending was performed. It also demonstrated antral gastritis, mild diffuse portal hypertensive gastropathy, and normal appearance of the duodenum. A random biopsy of the second portion of the duodenum was performed to rule out IgG4 deposition. Duodenal biopsy results showed IgG4-positive plasma cells, with no evidence of dysplasia or malignancy (Figure 2-hematoxylin and eosin stain of duodenal biopsy showing plasma cells, Figure 3-IgG4 stain positive on the duodenal biopsy).

**Figure 4:**
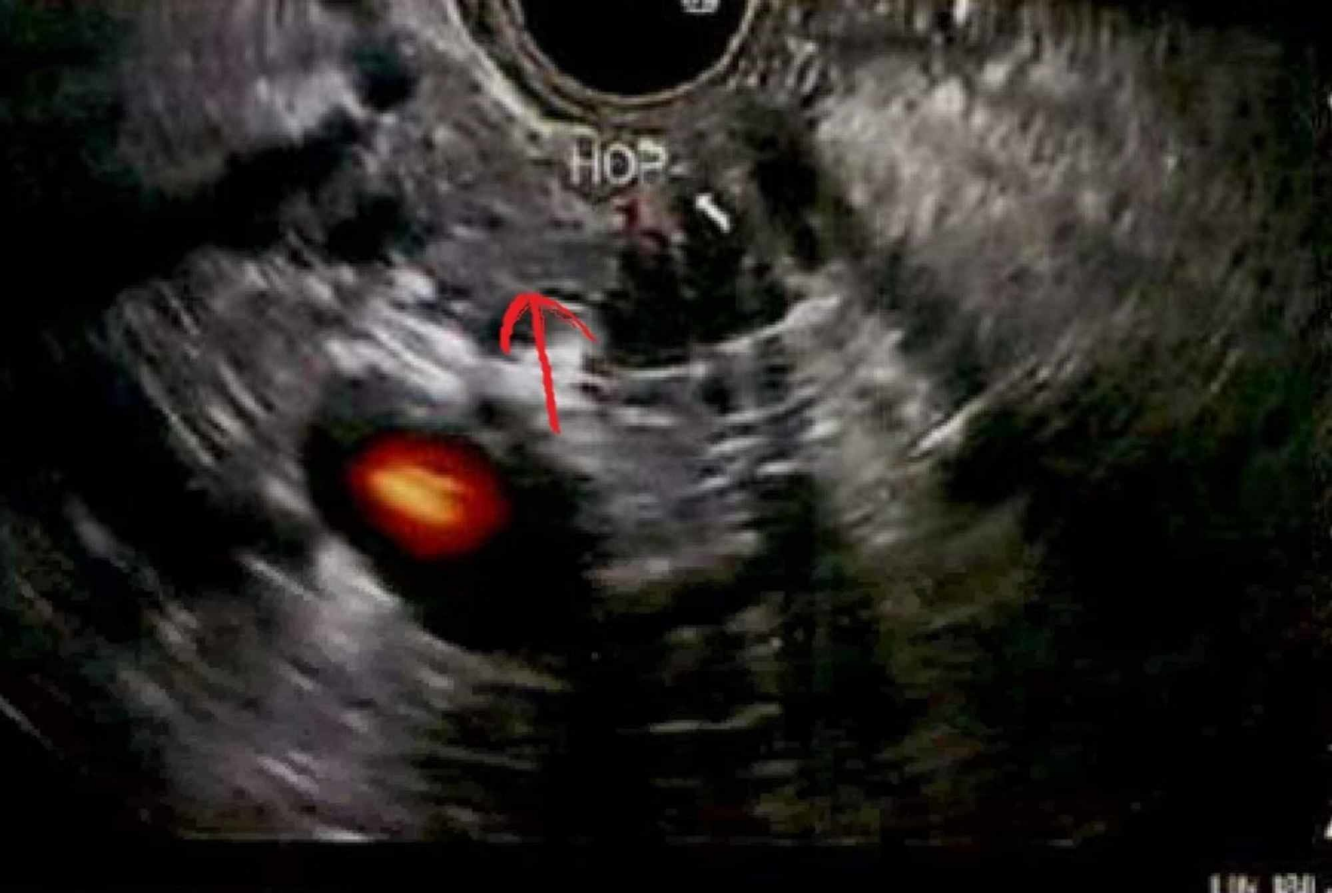
Endoscopic ultrasound showing hypoechogenic mass in the pancreatic body

Endoscopic ultrasound (EUS) demonstrated an irregular mass identified in the pancreatic body (Figure 4) and pancreatic tail mass which was hypoechogenic and measured 29 mm x 17 mm, with border poorly defined. Sonographic evidence suggested invasion into portal vein and splenic vein. There was dilatation of common bile duct by 10 mm, suggestion of stricture in the common bile duct, and lobular appearance of liver. However, there was no sign of significant endoscopic abnormality involving abdominal aorta or celiac trunk, and no celiac lymphadenopathy.

Pathology from the pancreatic head and tail mass showed fibrosis and inflammation suggestive of chronic pancreatitis. Given these findings, there was a concern for IgG4-RD, which was confirmed as IgG levels returned back elevated as follows: subclass 1 at 1,469 mg/dL subclass 2 at 1,027 mg/dL subclass 3 was normal, subclass 4 >300 mg/dL, and total serum globulin 3,127 mg/dL. The patient subsequently was started on prednisone and had received two months of systemic steroid therapy. Follow-up CT abdomen with contrast in two months showed improvement in the ill-defined area of decreased attenuation in the tail of pancreas; the overall appearance is most consistent with resolving pancreatitis, now measuring 1.9 x 1.5 cm. IgG levels decreased to the following levels: subclass 1 at 531 mg/dL (normalized), subclass 2 at 220 mg/dL, subclass 3 at 25 mg/dL (normalized), subclass 4 still elevated at 413.5 mg/dL (normal range 4-86), and IgG serum 1,155 mg/dL (normalized). Although there was an improvement in imaging with steroid therapy, IgG4 levels were still elevated; therefore, our patient was then started on rituximab therapy and received four doses of rituximab. Follow-up IgG4 panel showed significant improvement along with imaging evidence. IgG subclass 4 levels showed improvement to 164.4 mg/dL.

## Discussion

Pancreatic cancer is the fourth leading cause of cancer-related deaths in the United States [2]. Surgical resection is the only potentially curative treatment, but unfortunately, because of its late presentation, only up to 20% of patients are candidates for pancreatectomy [2]. The presenting symptoms of pancreatic cancer are usually vague. The most common presenting symptoms include abdominal pain, jaundice, and weight loss. Similar findings can also be seen in an entity such as IgG4-RD; thus, it is a challenge for many clinicians to differentiate both. However, it is crucial to distinguish both pancreatic cancer and IgG-RD affecting the pancreas because the clinical course, treatment plan, and prognosis are quite different.

IgG4-RD is a chronic multisystem immune-mediated disease that leads to tissue infiltration with IgG4-secreting plasma cells resulting in chronic fibrosis and scarring within the tissue which over time forms into a mass. It is a slow-growing process that usually remains indolent until it causes a mass effect or tissue infiltration. It can affect any tissue/organ within the body, and thus can have a variable presentation. The most commonly affected organs are the pancreas, lacrimal glands, salivary glands, kidneys, and arteries, and based on the organ affected, it can be known as many different names [1,3-6]. For example, with the involvement of the pancreas, it is known as type I autoimmune pancreatitis (IgG4-related pancreatitis) [3].

Patients generally present late as initially, they feel well and unlikely present as critically ill. However, due to the chronic inflammatory process, it can cause fatigue, weight loss, and obstructive jaundice, as well as vague abdominal pain if affecting the pancreas, as seen in our patient [7].

The diagnosis of IgG4-RD relies on clinical evaluation, laboratory testing, imaging, and histopathological findings. Serum IgG4 levels may or may not be present and are not required for the diagnosis of IgG4-RD, as can also be found to be normal in patients with IgG4-RD [1]. Thus, having normal IgG4 levels does not exclude the disease. The gold standard remains the formal biopsy.

However, if IgG4 levels are detected in patients with IgG4-related pancreatitis, they are usually significantly elevated in comparison with pancreatic cancer. Although mild elevation of IgG4 serum levels can be seen in pancreatic cancer [6], patients with IgG4-related pancreatitis may have serum levels of 663 mg/dL (median) vs. 51 mg/dL with pancreatic cancer [6]. Serum IgG4 concentration greater than 135 mg/dL results in a sensitivity of 95% and specificity of 97% when differentiating between pancreatic cancer and IgG4-related autoimmune pancreatitis [6]. High levels of serum IgG4 will usually give us a clue for IgG4-related pancreatitis; therefore, next, we can proceed with biopsy for confirmative pathological diagnosis.

Radiological studies such as CT/PET scans or MRI are essential in evaluating these mass-forming lesions based on the symptoms, although findings may not be as useful or help distinguish between pancreatic cancer and IgG4-related pancreatitis. The definitive diagnosis is still based on the formal biopsy with its characteristic histopathological findings that are identical in all of the involved organs. These include dense lymphoplasmacytic infiltrate with high levels of IgG4-producing plasma cells, storiform fibrosis, and obliterative phlebitis [4,8,9].

Open surgical biopsy is very invasive in obtaining a confirmative pathological specimen. The use of EUS-guided fine needle aspiration (FNA) can help evaluate a solid pancreatic mass, is less invasive, and can be useful in obtaining biopsy specimens for histopathological analysis [10]. As seen in our patient, initially, imaging findings demonstrated a mass in the pancreatic head and tail. Subsequently, EUS-guided FNA biopsies showed fibrosis and chronic inflammation as well as IgG4-positive plasma cells without any evidence of dysplasia or malignancy.

It is important to distinguish benign pancreatic masses from malignant cancer in the early phase as effective treatment can be administered as soon as possible because prognosis and treatment for both are quite different. Treatment for IgG4-RDs depends on the severity of symptom onset. The first line of treatment for IgG4-RD involves systemic glucocorticoids [8]. Subsequently, steroid-sparing agents such as azathioprine, methotrexate, cyclosporine, and even immune-modulators such as rituximab can be used as maintenance therapy [11-13]. Our patient showed good response with systemic steroids as evident by decreasing IgG4 plasma levels and decreasing the size of the mass lesions on follow-up CT scans. In addition to systemic steroids, our patient also received treatment with rituximab.

## Conclusions

Physicians should be familiar with the clinical presentations and diagnostic criteria of IgG4-related autoimmune pancreatitis resulting in a mass-like lesion vs. pancreatic cancer. An awareness of the differences between these two diseases may avoid misdiagnosis, prevent any unnecessary operative interventions, and result in prompt treatment.
